# Can traditional bonesetters become trained technicians? Feasibility study among a cohort of Nigerian traditional bonesetters

**DOI:** 10.1186/s12960-020-00468-w

**Published:** 2020-03-20

**Authors:** Ndubuisi Onu Onyemaechi, Ijeoma Uchenna Itanyi, Paulinus Okechukwu Ossai, Echezona Edozie Ezeanolue

**Affiliations:** 1 Department of Surgery, College of Medicine, University of Nigeria Ituku-Ozalla, Enugu, P.M.B. 01129 Nigeria; 2 Department of Community Medicine and Institute of Public Health, College of Medicine, University of Nigeria Ituku-Ozalla, Enugu, Nigeria; 3 Department of Public Health, Ministry of Health Enugu State, Enugu, Nigeria; 4 Institute of Maternal and Child Health, College of Medicine, University of Nigeria Ituku-Ozalla, Enugu, Nigeria

**Keywords:** Traditional bonesetters, Orthopaedic surgeons, Formal training, Feasibility

## Abstract

**Background:**

Traditional bonesetters (TBS) provide the majority of primary fracture care in Nigeria and other low- and middle-income countries (LMICs). They are widely patronized and their services are commonly associated with complications. The aim of the study was to establish the feasibility of formal training of TBS and subsequent integration into the healthcare system.

**Methods:**

Two focus group discussions were conducted involving five TBS and eight orthopaedic surgeons in Enugu Nigeria. Audio-recordings made during the focus groups were transcribed verbatim and analysed using a thematic analysis method.

**Results:**

Four themes were identified: Training of TBS, their experiences and challenges; perception of traditional bonesetting by orthopaedic surgeons; need for formal training TBS and willingness to offer and accept formal training to improve TBS practice. Participants (TBS group) acquired their skills through informal training by apprenticeship from relatives and family members. They recognized the need to formalize their training and were willing to accept training support from orthopaedists. The orthopaedists recognized that the TBS play a vital role in filling the gap created by shortage of orthopaedic surgeons and are willing to provide training support to them.

**Conclusion:**

This study demonstrates the feasibility of providing formal training to TBS by orthopaedic surgeons to improve the quality of services and outcomes of TBS treatment. This is critical for integration of TBS into the primary healthcare system as orthopaedic technicians. Undoubtedly, this will transform the trauma system in Nigeria and other LMICs where TBS are widely patronized.

## Background

In Nigeria and many developing countries, the treatment of diseases and injuries using traditional and cultural methods remain popular among the populace in spite of the availability of modern health care services [1, 2]. Traditional bonesetting is an age-long practice and has remained a part of health care delivery in many low- and middle-income countries (LMICs).

Traditional bonesetters receive no formal training in modern orthopaedic care, but mostly acquire informal training from family members as a part of ancestral heritage [3, 4]. Their practice of bonesetting is unregulated and lacks the basic scientific principles of fracture management as well as infection prevention and control [5]. Subsequently, the treatment of bone and joint injuries by the TBS has been reported to be associated with some complications such as mal-union, non-union, gangrene, chronic osteomyelitis, Volkmann’s ischaemic contracture and joint stiffness [6–8].

Despite the shortcomings in the training and outcomes of fracture treatment by the TBS, they enjoy high patronage and confidence in their communities [1, 5]. About 70–90% of primary fracture care is provided by the TBS in many rural communities in Nigeria [9, 10]. It is therefore of public health importance that this method of fracture treatment be recognized, formalized and regulated. The bonesetters appear to have met the fracture care needs of our rural communities for many decades prior to the advent of orthodox fracture treatment. Some of the reasons why they are widely patronized despite the availability of modern orthopaedic services include the following: socio-cultural beliefs, easy accessibility, relatively cheaper cost of treatment and perceived quicker services [1, 5, 11].

In Nigeria, both the traditional and orthodox fracture care methods have existed parallel to each for many decades. However, the relationship between the orthopaedists and TBS has been characterized by distrust and a sense of rivalry. While many TBS believe that the informal training they receive from their ancestors is superior to orthodox medicine, many orthopaedic surgeons in Nigeria believe that the TBS are untrainable [12]. Consequently, with the huge patronage enjoyed by the TBS in Nigeria and many LMICs, complications of fracture care ranging from limb- to life-threatening conditions have persisted and have remained a challenge to the orthopaedic surgeons practicing in these regions [6, 8].

Some studies suggest that TBS can be trained in safe methods of fracture treatment as a means of controlling these preventable complications [8, 12, 13]. However, the method and feasibility of this training has remained unclear with a paucity of reports on formal training of TBS. The aim of this study was to establish the feasibility and acceptability of formal training of TBS by the orthopaedic surgeons in Nigeria. It is believed that this training is the first step towards the regulation and integration of the TBS into the healthcare system as orthopaedic technicians. This will help to improve the collaboration between the orthodox and traditional fracture caregivers and bridge the gap between the two groups of practitioners with the ultimate goal of improving the outcomes of fracture treatment in Nigeria. This study may provide the template for the formalization and regulation of TBS practice in other LMICs where there are similar challenges with TBS practice.

## Methods

### Study setting

The study setting is Enugu State, located in the southeastern part of Nigeria. The state has a population of 3.2 million people [14], estimated at over 3.8 million in 2012. There are three tertiary hospitals that provide trauma care, all located in the capital city of the state, serving the state and other neighbouring states.

### Study design and data collection

This was a qualitative study, which collected data using focus group discussions. Focus group discussion (FGD) was appropriate because it is best suited for sharing experiences and perceptions among a similar group of participants. Furthermore, it allows for a richer and more flexible data collection that is not usually achieved with individual interviews while permitting spontaneity of interaction among the participants [15].

Two focus group discussions were conducted—one for traditional bonesetters (TBS) and one for orthopaedists on separate days. The TBS were recruited from their association, with the help of the Director of Public Health, State Ministry of Health. The FGD was held at the office of the Director of Public Health at the Ministry of Health, Enugu State. The orthopaedists were recruited from the three tertiary hospitals in Enugu namely: the National Orthopaedic Hospital Enugu, University of Nigeria Teaching Hospital Ituku-Ozalla and Enugu State University Teaching Hospital Enugu. The participants comprised both residents and specialists. A public health practitioner, trained in qualitative research methods facilitated the discussions as the moderator using a topic guide, developed from the research questions while a trained research assistant took notes. The sessions lasted between 60 and 75 min respectively. Written consent was obtained from all participants and the sessions were audio-recorded with participants’ permission. Identifiers were not used during the discussions to maintain confidentiality of the participants.

### Ethics approval

Ethical approval for the study was obtained from the Health Research Ethics Committee of the College of Medicine, University of Nigeria Ituku-Ozalla.

### Data analysis

The focus group discussion audio-recordings and notes were transcribed verbatim. Responses given in Pidgin English were translated into the English language. The anonymized transcripts were edited for clarity, grammatical errors and quality assurance. Data was analysed by thematic analysis. First, the transcripts were read at least twice to familiarize the researchers with the data. Then the research team collaboratively developed a coding scheme by sorting the data into categories and sub-categories. Sub-themes of related categories were grouped into central themes. Central themes, sub-themes and emerging themes were generated from the data and the topic guides. The data were analysed according to themes generated from the transcripts and by relating outstanding points in the responses and analytic concepts to the objectives of the study. Phrases with special connotations and keywords were noted in the transcripts and presented as illustrative quotes. The rationale for adopting a step by step approach was to ensure we did not miss out any concept in the data.

## Results

### Characteristics of the participants

Three male and two female traditional bonesetters participated in the FGD. Their mean age was 40.6 + 3.2 years, and their median years of practicing experience was 10 years. Most of the TBS participants practised in urban areas of the state and had a secondary level of education. The number of fracture patients seen monthly was 10 + 2.

Seven out of the eight orthopaedic surgeons were males. The majority of them were senior residents. Their mean age was 41.6 + 4.5, and the mean years of orthopaedic practice were 14 + 6 years.

We identified four themes as presented below.

### Type of training received by TBS, their experiences and challenges

All the TBS reported that they did not receive any formal training in their bonesetting practice. They saw traditional bonesetting practice as an inheritance or a gift from God. They also stated that there was no specified period of training, rather, they acquired their skills by watching their relatives who were practicing traditional bonesetting. One participant reported learning his skills from a TBS who was not a relative. All the participants admitted that they were not certified and their practice was not regulated. The participants shared the following:

“This is an inheritance from God. I did not learn anything from anybody. I didn’t want it before but the thing was disturbing me since how many years now. That’s why I started, even the medicine, everything, is my own.” (Participant 3, TBS focus group)

“This ASTRABON (Association of traditional bonesetters of Nigeria), I learnt it from my mother when I was small, then when I grew up like this, I started it.” (Participant 2, TBS focus group)

“…I was very close to my daddy and my grandma when they were doing all those things and from there, I was picking up; and studied along *and trained along and that is where I found myself.”* (Participant 4, TBS focus group)

When asked about their experiences with traditional bonesetting practice, all the TBS reported that they recorded lots of successes in their practice. All participants stated that they were able to treat cases that could not be handled by orthodox doctors.

“There are many severe cases that have been brought, severe cases that hospitals could not handle that in my own very eyes, we have handled them. The sweeter part of it is testimonies we get, that a patient will come, leg or hand or any part of the body broken, at the end of the day, within the space of three weeks, one month the person is walking on his own without holding any staff, without anybody supporting the person. It gives you joy as a practitioner. The person comes with testimonies, with gifts; no matter how little, appreciation is appreciation”. (Participant 4, TBS focus group)

Only one of the participants acknowledged that he had experienced some failures in his practice. However, he highlighted that he recorded more successes than failures. The other participants did not accept that they had experienced any failures in their practice.

“In our practices, we see failures; not one not two and not three.…. Yes, even in orthodox practices, there is failure as well as in traditional bone healing”. (Participant 4, TBS focus group)

The participants shared some of the challenges they encountered in their practice. The most notable challenges were lack of basic infrastructure and equipment and lack of or incomplete payment by patients. It was reported that the government had given them a building, which needed to be completed and equipped. They all agreed that some basic equipment would make their work easier.

“They haven’t plastered the building, no window, no door. We need to plaster that place, put hospital equipment like crutches, beds and eeeh, the main thing is massaging machine which we use to massage the bones.” (Participant 2, TBS focus group)

Every TBS reported that lack of or incomplete payment for their services was a major challenge in their practice. Some reported using their money to buy food or drugs for the patient after which the patient’s *relatives would plead to be given enough time to pay.*

“There are people who don’t have money and due to pity, you will use your own money to buy what you will use to do the work. When you finish buying those materials, you will do the work, the patient will not give you money. The person will tell you that there is no money, you won’t kill the person, you will leave that person. So, but we will be doing what we ought to do as human.” (Participant 3, TBS focus group)

### Perceptions of traditional bonesetting by orthopaedic surgeons

Most of the orthopaedists felt that traditional bonesetters were an inevitable part of society and were filling a gap caused by the shortage of orthopaedists in Nigeria. However, all the orthopaedists expressed that although the traditional bonesetters were filling a gap, they were causing a lot of havoc due to the huge complications associated with their practice. They noted that most of the complications resulted from mismanagement of fractures, particularly the application of splints that were too tight.

“They are filling the vacuum, because there are so many places where they don’t have even a doctor. They don’t even have a general practitioner, not to even talk of specialist or somebody who specialized in orthopaedics. In those places, if somebody has a fracture, where does the person go? There is usually somebody who attends to them. So my perception of them is that they are filling a gap or a vacuum that has been created by lack of orthopaedists. The truth is we don’t have enough orthopaedists in the country, how many do we have? Only about Four hundred and something are registered… ” (Participant 8, orthopaedist focus group)

“With respect to how we see them, they are indeed causing a lot of damage. They have caused more harm than good.” (Participant 2, orthopaedist focus group)

Two of the orthopaedists shared a personal experience of mismanagement by TBS.

“I am also a victim. I can remember growing up many years ago when I was playing as a young boy climbing a mango tree, I fell down and had a fracture on my left hand. My parents took me to a traditional bonesetter and today I still see the deformity. I had a mal-united styloid process. If I do anything heavy with it, I still feel the pains and it reminds me of that problem.” (Participant 2, orthopaedist focus group)

“In fact, there is the case of my nephew, just about 3 months ago, he had his leg amputated for a closed fracture following TBS mismanagement, so my own personal experience is more of worry, what can we do to ameliorate the impact of this mismanagement?” (Participant 1, orthopaedist focus group)

All the orthopaedists perceived the patronage of TBS as huge. Six of the eight orthopaedists believed that patronage of TBS cuts across all educational levels.

“Their patronage is massive because even those that present to the accident and emergency here, most times after resuscitation, some of them leave against medical advice and the percentage that discharge against medical advice is quite enormous even though this place is a national trauma centre. If you survey the population, you will see that more than 95% of them patronize the traditional bone setters.” (Participant 6, orthopaedist focus group)

The major reasons the orthopaedists expressed for the huge patronage of TBS were a lack of health insurance and huge out-of-pocket payments and cheaper cost of TBS treatment compared to orthodox fracture treatment, including the flexibility of payment in instalments; strong cultural belief system and trust by society; and easy accessibility of TBS compared to orthopaedists.

“Actually, it is very worrisome, with this problem of healthcare that is not affordable, people pay out of their pockets, and most times when they come to hospital, they feel the bills are too high and they get it cheaper at the bone setting place. Can you imagine a treatment session for a week at 5,000 Naira, which is not possible in a government hospital?” (Participant 6, orthopaedist focus group)

“The society even views them closer to them than even us the doctors maybe because they can easily access them without any payment of hospital card ….” (Participant 3, orthopaedist focus group)

“Another reason why their patronage is very high is that they tell the patients that once you go to the hospital, they will cut your leg.” (Participant 5, orthopaedist focus group)

On the contrary, all the TBS viewed orthopaedists as feeling superior. They shared their experiences with treating cases that orthopaedists could not treat. All but one of the participants expressed the view that their practice was superior to orthopaedic practice because it is natural and older than orthodox practice. They also expressed their displeasure with orthopaedic practice, particularly the use of POP and metal implants.

“Any work that orthodox handle and they know that they cannot do the work, they still refer the work to us, traditional practitioners.” (Participant 1, TBS focus group)

“You know ours is natural, natural blend….from the soil, originally made by God.” (Participant 4, TBS focus group)

“So, for the orthodox doctors and traditional doctors, we are the first people God created.” (Participant 2, TBS focus group)

### Need for training of TBS and their integration into the primary healthcare system

The traditional bonesetters expressed the desire for a TBS training centre or school to be established and they felt this would formalize their training and enhance their recognition by the government and the populace.

“We bone healers, we need to have our own school for traditional bone healers because we have experts who can come and teach in that place.” (Participant 2, TBS focus group)

When asked about specific areas where they need training to improve their skills, one of the traditional bonesetters expressed the need for training in modern science and use of some hospital equipment that they could use in their practice.

“There’s an idea from science that we may not know, a time can come for a seminar to be organized for us or it can be in a class where a professor can come and teach. There are some of the machines hospitals use that we may need. We can only be trained on how to handle them, like testing machine. So those things, we may not have personal experience of how to handle them but we can be taught.” (Participant 4, TBS focus group)

Conversely, all the orthopaedists emphasized the need for education of TBS to limit complications in their practice. The recommended areas of training the orthopaedists highlighted were early recognition of cases that TBS should not treat, otherwise called “patient selection”, and splinting techniques. They remarked that focusing on the commonest complications seen from TBS practice, notably gangrene resulting from splints that are too tight, would be very helpful. The need for early referral was also emphasized.

“…my area of emphasis is on patient or client selection. It will go a long way to limit the damage. If we are able to get that one right, then we can step up to the things they should do during their practice but patient selection is the first, I think that is where the friendliness should start. If we start by telling them what to do in their practice, they will tell you that their inspiration comes from the spirit.” (Participant 1, orthopaedist focus group)

“I wouldn’t even say they should manage this or manage that because it is not a regulated practice. It is very difficult to limit what they should treat or not.....so if we can just focus on the things that cause the most harm – amputations which can be prevented by minimizing how tight the splints are and avoiding managing fractures that have open wound because they don’t have sterile materials, we will achieve a lot.” (Participant 8, orthopaedist focus group)

The method of training suggested by the orthopaedists was instructional training and use of pictorials. They felt that use of pictures of some complications from TBS practice and proper ways the cases could have been managed would be effective.

“Pictures of various complications can be displayed as well as how they could have been managed to prevent such complications….something very simple in the kind of pictorials and they can go home with it.” (Participant 7, orthopaedist focus group)

Six of the eight orthopaedists supported integration of TBS into the primary healthcare system. They suggested the establishment of a certification board for TBS by the government to identify, certify and regulate the practice of TBS. It was noted that because of the scarcity of orthopaedists and the huge patronage of the TBS, there was a dire need to give them a legal platform and draw them closer to the health system in order to improve their practice and ultimately improve health outcomes. One of the participants gave an example with obstetricians and traditional birth attendants, and how integration and training of the latter has improved birth outcomes. Some of their responses were:

“Integrating them into the primary healthcare system will be a good idea considering their huge patronage. Instead of making them illegal and they remain in the dark causing havoc, it is better they are given a legal platform. For the legal platform to stand, they must have a control board so they can be sued because without control, you can’t hold them liable. Such a control board will be able to regulate their practice so they can become part of the primary healthcare system.” (Participant 1, orthopaedist focus group)

### Willingness to offer and to accept formal training to improve TBS practice

All the orthopaedists expressed their willingness to offer training to TBS to improve their practice and reduce complications. However, three of the participants suggested that such training should occur in a neutral environment to avoid misunderstanding between the two groups of practitioners.

“So if there should be any kind of training, it should happen in a neutral place, a place that is neither in our hospital nor where they are practicing.” (Participant 8, orthopaedist focus group)

A participant expressed concern about the willingness of TBS to accept training to improve their practice, and advised that the opinions of the TBS be sought.

“We need to be sure that the TBS are willing to accept training or education to improve their practice. We need to hear from them too.” (Participant 4, orthopaedist focus group)

On the other hand, one of the TBS expressed his willingness to accept training support from orthopaedists. He shared the possibility of a deficiency in knowledge of TBS, which could be filled by practitioners in orthodox practice. He also suggested a way to provide such training to be acceptable to TBS. His opinion was passively supported by the other TBS.

“Now if anybody thinks that he has known it all, that there’s no room, no space for learning anything, that will not be us; that will not be a person like me, we learn every day. So, we need…, the people that read wide, went to universities, have Masters, PhD, there are things they gathered we may not know….We need to know, knowledge is power. We need to get to such knowledge. So, we really need to know more based on science, things we learn from reading…..We need to know more, you can organize a seminar for us. I think now, the better way is, you people have been doing it like this and have been achieving success, I think if you add this, you will achieve greater success.” (Participant 4, TBS focus group)

## Discussion

All the traditional bonesetters acquired their skills through informal training by apprenticeship mostly from relatives and family members. One of the major shortcomings of the practice of the traditional bonesetters in Nigeria and other LMICs is their process of skills acquisition in bonesetting. Our study corroborates previous reports that the training of the TBS is informal, undocumented and non-standardized [1, 3]. The training process is also associated with secrecy and hoarding of information from non-family members because it is seen as part of an ancestral heritage [16, 17]. Consequently, this may be associated with a decrease in the quality of information and skills passed on to learners over many generations.

Additionally, the training is passed on by verbal communication, and there are no peer-review mechanisms, continuing education programs or any regulations. The quality of the training cannot be guaranteed, thus making the practice non-standardized and prone to complications [8, 18]. It is imperative to have a formalized, standardized and regulated training of TBS. A potential solution is a competency-based training method with certification in the form of micro-credentials. This will guarantee safe, satisfactory and predictable outcomes of fracture treatment by TBS in many LMICs where a majority of primary fracture treatment is provided by them.

Bridging the gap between the orthodox practitioners and TBS will ensure a more effective fracture treatment in developing countries. Therefore, the perceptions of both practitioners of each other are critical. All the participants (orthopaedists) in our study recognized that the bonesetters play a vital role in filling the gap created by the shortage of orthopaedic surgeons in Nigeria and need to be equipped to render safer services. With a population of over 180 million people, Nigeria has approximately 400 orthopaedic surgeons. The density of orthopaedic surgeons in Nigeria is 0.22 per 100,000 population. This is very low compared to 9.2 per 100,000 population in USA [19] and 6.9 per 100,000 population in UK [20]. The majority of the orthopaedic surgeons practise in the tertiary hospitals in the cities and urban areas with little or no presence in the rural communities. This dearth of orthopaedic surgeons particularly in the rural communities in Nigeria may be contributing significantly to the huge patronage of TBS in Nigeria.

All the orthopaedists recognized the huge patronage of TBS compared to orthodox fracture treatment among the populace which cuts across educational and socio-economics status. This is not surprising because, with the gross shortage of orthopaedists, the only alternative is the TBS who are more accessible to the rural dwellers. The reasons cited for the huge patronage of TBS in this study are similar to reports from other studies [1, 5, 11]. Notably, the complications associated with TBS practice were considered a major problem by the orthopaedists. The exact complication rates of TBS practice are not known because only the patients with complications present to the orthopaedists for treatment. Since not all the patients treated by TBS present to the orthopaedists, it is believed that many patients with undisplaced or minimally displaced fractures may have been successfully treated by them. It was noteworthy that the orthopaedists did not express concern about the TBS taking over their work; rather, they were mostly concerned about the safety and risks associated with TBS practice. Overall, the collaboration between the orthodox and traditional fracture caregivers will bridge the age-long rivalry and distrust that has existed between these two groups of practitioners.

Interestingly, majority of the TBS participants felt their practice was superior to orthodox practice. They claimed that their practice is natural, predates orthodox practice and was originally made by God. Traditional medicine practitioners were in practice long before orthodox medicine was introduced to developing countries [21]. The first orthodox hospital in Nigeria was built in 1873 [22]. However, prior to this time, traditional medicine was the only available healthcare service in Nigeria. This long-standing history and the deep cultural acceptance of traditional medicine in Nigeria and other developing countries seem to have given the TBS the feeling of superiority over modern medicine. Consistent with the findings in this study, communities perceive traditional medical practitioners as members of their communities, see them as more accessible and trust them more than orthodox practitioners.

We noted that the bonesetters recognized the need to formalize their training and skill acquisition process. According to them, this will enhance their recognition by Government and the populace. Their request for a training school and for seminars to be organized for them express their desire for formal training in modern medicine to improve their knowledge and skills. This observation is critical in the bid to formalize the training and skill acquisition by TBS. One of the TBS expressed willingness to accept training support from orthopaedists, which was passively supported by the others. He recognized the possibility of a deficiency in knowledge and skills among the TBS which could be addressed by training by orthodox practitioners. This observation is in contrast to opinions expressed in previous reports that the TBS are untrainable and should not be offered any opportunity to improve their knowledge and skills [12]. It was remarkable that the TBS were interested in seeking formal recognition by government. This provides a great opportunity for micro-credentialing of all the TBS practitioners who complete the proposed competency-based training.

All the orthopaedists in this study believed that the TBS are trainable and require further training. The need for training is to improve the knowledge and skills of the bonesetters. They were also willing to offering this training to the TBS. On the basis of the type of complications from TBS practice that present to them, the orthopaedists recommended specific areas that the training should address. These areas included patient selection, splinting techniques and a referral system. It is believed that the most dreaded complications from TBS practice such as limb gangrene, septicemia, tetanus, chronic osteomyelitis and Volkmann’s ischaemic contracture could be controlled by these interventions. The proposed method of education is instructional training using pictures and practical demonstrations. This was considered appropriate because of the level of education of most bonesetters. The majority of the orthopaedists support the integration of TBS into the primary healthcare system after formal training and registration by a regulatory board. This observation corroborates the recommendations of previous authors that training of bonesetters was a means of improving the outcome fracture treatment in developing countries [8, 12, 13]. The findings from this study may be potentially applicable to other areas of traditional medical practice. For instance, the traditional birth attendants (TBAs) may be formally trained to provide skilled obstetric care in the rural communities, thereby improving maternal and neonatal outcomes.

## Conclusion

Traditional bonesetters in Nigeria receive no formal training in bonesetting. However, they receive huge patronage from the populace mainly due to lack of access to modern orthopaedic services. The feasibility and acceptability of formal training of traditional bonesetters demonstrated in this study has provided the opportunity for improving the knowledge and skills of the TBS. Both the orthopaedists and the TBS admit that standardized and formalized training will ultimately improve the quality of services and outcomes of their fracture treatment by TBS. The integration of the trained TBS into primary healthcare system as orthopaedic technicians will transform the trauma system in Nigeria and other LMICs to provide culturally acceptable and effective fracture treatment.

## Data Availability

The datasets used and/or analysed during the current study are available from the corresponding author on reasonable request.

## Abbreviations

TBS: Traditional bonesetters
LMICs: Low- and middle-income countries
FGD: Focus group discussion
PhD: Doctor of Philosophy
USA: United States of America
UK: United Kingdom
TBA: Traditional birth attendants
